# Reply to Kapur, V. Is Pre-Exposure Prophylaxis a Cost-Effective Intervention to Avert Rabies Deaths among School-Aged Children in India? Comment on “Royal et al. A Cost-Effectiveness Analysis of Pre-Exposure Prophylaxis to Avert Rabies Deaths in School-Aged Children in India. *Vaccines* 2023, *11*, 88”

**DOI:** 10.3390/vaccines11040807

**Published:** 2023-04-06

**Authors:** Abhishek Royal, Denny John, Omesh Bharti, Ritesh Tanwar, Deepak Kumar Bhagat, Retna Siwi Padmawati, Vishal Chaudhary, Reddicherla Umapathi, Pradeep Bhadola, Adi Utarini

**Affiliations:** 1 Faculty of Medicine, Public Health and Nursing, Universitas Gadjah Mada, Yogyakarta 55281, Indonesia; 2 Faculty of Life and Allied Health Sciences, Ramaiah University of Applied Sciences, Bengaluru 560054, India; 3 State Institute of Health and Family Welfare, Department of Health & Family Welfare, Government of Himachal Pradesh, Shimla 171009, India; 4 Directorate of Health Services, Government of Madhya Pradesh, Bhopal 462002, India; 5 Centre of Social Medicine and Community Health, Jawaharlal Nehru University, Delhi 110067, India; 6 Department of Physics, Bhagini Nivedita College, University of Delhi, Delhi 110021, India; 7 Department of Biological Sciences and Bioengineering, Inha University, Incheon 22212, Republic of Korea; 8 Centre for Theoretical Physics and Natural Philosophy, Mahidol University, Nakhonsawan Campus, Phayuha Khiri, NakhonSawan 60130, Thailand

Thank you so much for forwarding the critical analysis the author (VK) conducted on our recently published modelling study ‘A Cost-Effectiveness Analysis of Pre-Exposure Prophylaxis to Avert Rabies Deaths in School-Aged Children in India’ in your reputed journal [1]. We are thankful to the author for this analysis and for providing his input [2]. 

The current scenario of human rabies surveillance in the states and its reporting to the central government is poor in India [3]. The reported data are institution-based/passive surveillance, with inherent limitations of the iceberg phenomenon of a disease in a community/population. Moreover, a concordance of 43% on dog bite data was reported between the Integrated Disease Surveillance Programme (IDSP) and the Association for Prevention & Control of Rabies in India (APCRI) survey [3,4,5]. The numbers on the animal bite incidence and burden of rabies in the literature and government data are highly under-reported [3]. 

There have been reports from various field-based studies on the exponential increase in animal bites, especially in hilly and forest areas. The state of Himachal Pradesh reports an exponential rise (almost six times) in cases of dog bites from 11,412 in 2013 to 65,906 in 2021 through IDSP (as reported by IDSP, Himachal Pradesh). A recent publication reported a consistent rise in the cases of dog bites and rabies deaths in the state of Kerala, with more than 200,000 cases of dog bites and 21 rabies deaths (almost double the deaths reported in the previous year) in 2022 [6]. Moreover, six out of these 21 victims died despite of administration of rabies immunoglobulins and anti-rabies vaccination. The neighbouring state of Tamil Nadu also reported an alarming number of rabies deaths during the first eight months of 2022 [6]. 

In 2021, Dr Shewale and others from the Division of Zoonotic Diseases Programme, National Centre for Disease Control, Ministry of Health & Family Welfare, India, published a short communication on PrEP against rabies for high-risk groups [7]. This paper reported that 13 states had shown more than 5% of the incidence of animal bites among children under 18 years of age [7]. The paper also proposed that PrEP should be piloted for street children in high-prevalence districts. Moreover, the communication also supported the exploration of cost-effectiveness studies and inputs from relevant stakeholders/expert groups for the government to consider pre-exposure prophylaxis of all children in selected states/districts with a high prevalence of animal bites [7]. 

Our decision tree model was adapted from a previously published model developed to compare rabies pre-exposure prophylaxis with post-exposure prophylaxis in children in the Philippines [8]. An in-depth literature search using a published systematic review was conducted to explore relevant data inputs for this modelling study. The data extracted included an independent review of grey and published literature, nationally representative (WHO-APCRI) surveys, programmatic reports, and national and state databases for data inputs for this modelling study. As mentioned in our paper, the literature reporting the relevant data points from the most recent, community-based studies conducted on the target population was preferred for this modeling study. Additionally, the model, its data inputs and assumptions were validated by the field experts working on implementing rabies interventions in India. The model development, data inputs, and analysis adhered to the ISPOR-Society for Medical Decision-Making Best Practices for Modeling [9].

The reflections presented on the data inputs in the letter are as follows:

1. Annual Bite Incidence

Our model was constructed for a cohort of children in the age group of 5–15 years. The paper, i.e., Sudarshan et al. 2006, cited by the author (VK), was published in 2006 and was derived from the WHO-APCRI survey conducted in 2003–2004 [5]. This paper reported an annual bite incidence of 2.5% in a cohort of children under 14 years of age (including children in the cohort of 0–5 years). Since the data reported in Sudarshan et al. (2006) paper are over 15 years old and represent a population that differs from the cohort considered in our study, the proposed data input was not included in our model. Agrawal et al. (2015) reported data in the cohort of 5–14 years of age group from a community-based cross-sectional survey [10]. Therefore, this data input with a bite incidence of 4.4% was considered for our model.

2. Probability of bite from a rabid animal

The community survey conducted as a part of the WHO-APCRI survey (2017) reported that only 26 dogs (of the total number of 54 cases of animal bites) were available for a follow-up and alive after an observational period of 10 days [4]. Since the follow-up rate was less than 50% and cannot be used as a substitution for a confirmed rabid animal, it was not taken as a replacement for this data input. As already stated in our paper, this was an assumption and is taken to be 0.295 as reported in the health facility survey of WHO-APCRI survey 2017 [4]. The survey mentions explicitly that “29.5% of the biting animals (n = 529) showed some signs of suspected rabies such as aggression, hypersalivation, biting other animals and changes in dog bark; but none of the biting animals was proven to be rabid” [4]. Moreover, it also mentions that “among the biting animals, only 31 dogs/cats were followed-up due to logistical reasons/feasibility for 14 days by the veterinary team to know the rabid status of the biting animal” [4]. Furthermore, in another study, among 300 samples taken from dogs, 168 samples (56%) were found to be positive for rabies, compared to 32% in a similar study conducted in 2016 [6].

There are limited data on the probability of a bite from a rabid animal from India. The follow-up of biting animals and confirmation of rabies is challenging, and the data on confirmed animal cases are scarce. Therefore, after discussing with rabies experts in India, this assumption was considered the data point in the study.

3. A one-way sensitivity analysis was conducted to test the uncertainty of the input variables on the outcomes of all the strategies tested in this modeling study. The upper and lower limits of data inputs were extracted from the literature for this study. The limits were set at varying degrees of variation as per the feasibility of values for the variables where additional data inputs were not available in the literature. The paper has already mentioned that the outcome of the study is sensitive to the variables on rabies positivity of the biting animal and animal bite incidence.
4. The limitations of the modelling study are clearly mentioned in the paper. We accept that there is a lack of nationally representative, age-specific data on animal bites and rabies in India. We have also disclosed the various assumptions taken due to the dearth of literature. The data inputs were extracted from the closely representative studies and were validated by the field experts.
5. The paper has been concluded with a suggestion to conduct studies in primary settings to extract data useful for future cost-effectiveness studies of shorter regimens in real-time settings. The shorter regimen of PrEP has been proven to be cost-effective in our model. As with any health economic model, it is always advised to extract data inputs from real-time settings and re-run the analysis for more realistic outcomes for any future cost-effectiveness analysis.

This cost-effectiveness analysis was conducted and reported following the CHEERS checklist [11]. While we accept the critical analysis conducted by the author, the modelling results should be discussed and viewed as a combination of data inputs, results, conclusions, and recommendations. Our paper should be seen as detailed evidence along with the changing epidemiology of bite cases and as first of its kind study in India that attempted to conduct a cost-effectiveness analysis on a pertinent issue to suggest an intervention to avert painful deaths in school-aged children in India. We understand that a dearth of literature has been mentioned in the limitation section of our paper. 

There is limited evidence on the PrEP intervention to avert rabies deaths among children in India. Our evidence adds to the current evidence and would be useful for policy discussions on the management of rabies bites using pre-exposure prophylaxis in the country.

## Data Availability

This study is based on a decision tree modelling adapted from the existing models used in the estimation of the global burden of rabies, and the data inputs are extracted from the existing literature or programmatic data/national surveys.
